# Dissociation of Detection and Discrimination of Pure Tones following Bilateral Lesions of Auditory Cortex

**DOI:** 10.1371/journal.pone.0044602

**Published:** 2012-09-05

**Authors:** Andrew R. Dykstra, Christine K. Koh, Louis D. Braida, Mark Jude Tramo

**Affiliations:** 1 Program in Speech and Hearing Biosciences and Technology, Harvard-MIT Division of Health Sciences and Technology, Cambridge, Massachusetts, United States of America; 2 The Institute for Music and Brain Science, Auditory Neuroscience Program, Department of Neurology, Massachusetts General Hospital and Harvard Medical School, Boston, Massachusetts, United States of America; 3 Research Laboratory of Electronics, Massachusetts Institute of Technology, Cambridge, Massachusetts, United States of America; Hotchkiss Brain Institute, University of Calgary, Canada

## Abstract

It is well known that damage to the peripheral auditory system causes deficits in tone detection as well as pitch and loudness perception across a wide range of frequencies. However, the extent to which to which the auditory cortex plays a critical role in these basic aspects of spectral processing, especially with regard to speech, music, and environmental sound perception, remains unclear. Recent experiments indicate that primary auditory cortex is necessary for the normally-high perceptual acuity exhibited by humans in pure-tone frequency discrimination. The present study assessed whether the auditory cortex plays a similar role in the intensity domain and contrasted its contribution to sensory versus discriminative aspects of intensity processing. We measured intensity thresholds for pure-tone detection and pure-tone loudness discrimination in a population of healthy adults and a middle-aged man with complete or near-complete lesions of the auditory cortex bilaterally. Detection thresholds in his left and right ears were 16 and 7 dB HL, respectively, within clinically-defined normal limits. In contrast, the intensity threshold for monaural loudness discrimination at 1 kHz was 6.5±2.1 dB in the left ear and 6.5±1.9 dB in the right ear at 40 dB sensation level, well above the means of the control population (left ear: 1.6±0.22 dB; right ear: 1.7±0.19 dB). The results indicate that auditory cortex lowers just-noticeable differences for loudness discrimination by approximately 5 dB but is not necessary for tone detection in quiet. Previous human and Old-world monkey experiments employing lesion-effect, neurophysiology, and neuroimaging methods to investigate the role of auditory cortex in intensity processing are reviewed.

## Introduction

By the close of the 20th century, it seemed reasonably well-established on the basis of neuropsychology studies in patients and selective-ablation experiments in animals that auditory cortex was devoted to higher functions such as pattern recognition but played little or no role in elementary functions involving discrimination of a single acoustic feature and its corresponding percept (e.g., pure-tone intensity and loudness; for review see [1], [2]). However, experiments with neurological patients employing rigorous psychoacoustic methods and in vivo lesion localization have since demonstrated that lesions of auditory cortex impair pure-tone frequency processing and pitch discrimination, even when pure-tone audiograms are within normal limits [3]–[6]. These observations suggest a dissociation between the effects of auditory cortex lesions on auditory sensation (i.e., detecting the presence of a sound) and auditory perception (i.e., determining whether and how two sounds differ in one or more attributes).

The present study focuses on pure-tone intensity processing in relation to tone detection and loudness perception. The loudness of a sound source and its change over time conveys information about its size, location, movement, significance, and identity. Humans and animals modulate the loudness of vocal communication sounds to convey meaning and emotion, and musical dynamics is a key ingredient of musical aesthetics. Normal adults demonstrate remarkably high perceptual acuity for loudness changes under optimal listening conditions: the just noticeable difference (jnd) for two-tone loudness discrimination is less than 1 dB at moderate and higher intensities throughout almost the entire audible spectrum (for review see [7]). Given recent evidence that auditory cortex supports high perceptual acuity for pure-tone pitch perception [3], [6], we hypothesized that it also subserves fine-grained loudness perception.

This idea is supported by recent studies utilizing functional neuroimaging which showed increased fMRI activation in the auditory cortex with increasing sound level (see, e.g., [8]–[11]), particularly in posteromedial portion of the tranverse gyrus of Heschl (TG) [8], [11], the presumed core area of human auditory cortex [12]–[14]. However, such correlational studies cannot establish the necessity of a given structure for a particular function, and psychoacoustic experiments with neurological patients who have bilateral auditory cortex lesions provide strong tests of hypotheses about the functional role of human auditory cortex in auditory sensation and perception. While the rarity of cases with bilateral lesions limits the inferences one can draw about structure-function correlates in the general population, single-case studies provide an important means of establishing existence proofs.

This paper reports the results of a series of original experiments examining loudness perception in a population of normal adults and a middle-aged, mixed-handed man, Case A1+, with chronic, bilateral middle cerebral artery (MCA) infarcts that include all of left and right primary auditory cortex (A1) and much of auditory association cortex (AA). We predicted that: 1) jnd’s for tone loudness discrimination (“louder”-“softer” judgments) would be greater in Case A1+ than normals; 2) his jnd's would be greater in the ear contralateral to the larger (right MCA) lesion; and 3) his tone loudness discrimination would be impaired out of proportion to tone detection.

## Materials and Methods

### Ethics Statement

All procedures were approved by the Institutional Review Boards at the Massachusetts General Hospital (MGH) and the Massachusetts Institute of Technology (MIT), and written informed consent was obtained from all participants prior to their participation.

### Participants

#### Case A1+

Case A1+ is a 46-year old mixed-handed man who suffered ischemic infarcts in the distribution of the right middle cerebral artery in 1980 and the left middle cerebral artery in 1981. He has twelve years of education and is not trained in music performance or theory. At the time of the present experiments, he was the primary caretaker of his and his wife's three young children and was on warfarin anti-thrombotic therapy and phenytoin anti-convulsant therapy.

**Figure 1:**
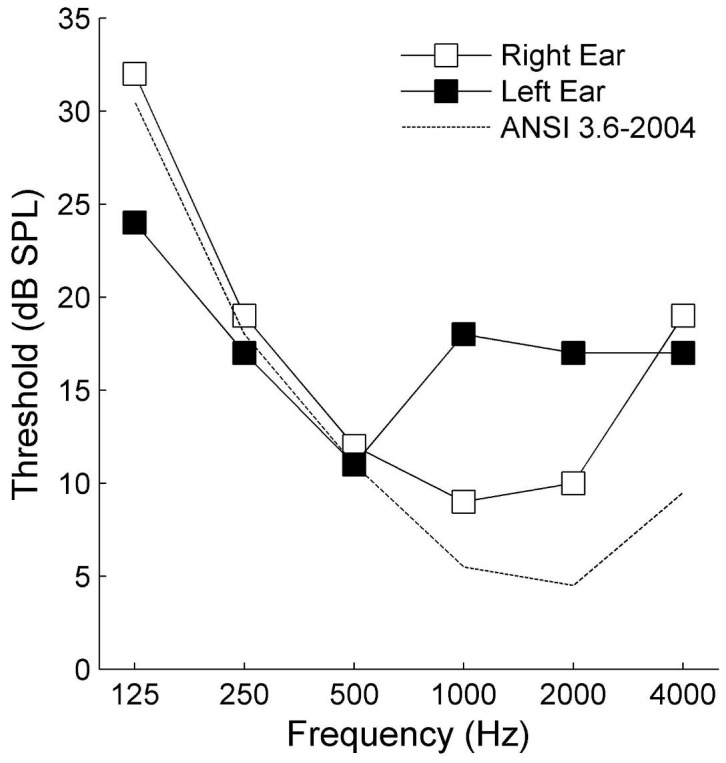
Case A1+ pure-tone audiogram for left-ear (filled symbols) and right-ear (open symbols) presentation. Clinically-defined normal limits extend 25 dB above the dotted line, which indicates the average threshold from the ANSI standard.

Details of the clinical history, neurological and audiological examinations, and radiographic findings have been reported previously [4], [6], [15], [16]. In brief, the first cardioembolic stroke presented with left hemiplegia and left hemisensory loss; the second presented only with complete loss of hearing. Detection of tones and natural sounds at moderate and high intensities returned within a month, but perception of speech, music, environmental sound, and sound source location remain impaired. At the time the current study was conducted, Case A1+ had thresholds which were within clinically-defined normal limits (Fig. 1).

**Figure 2:**
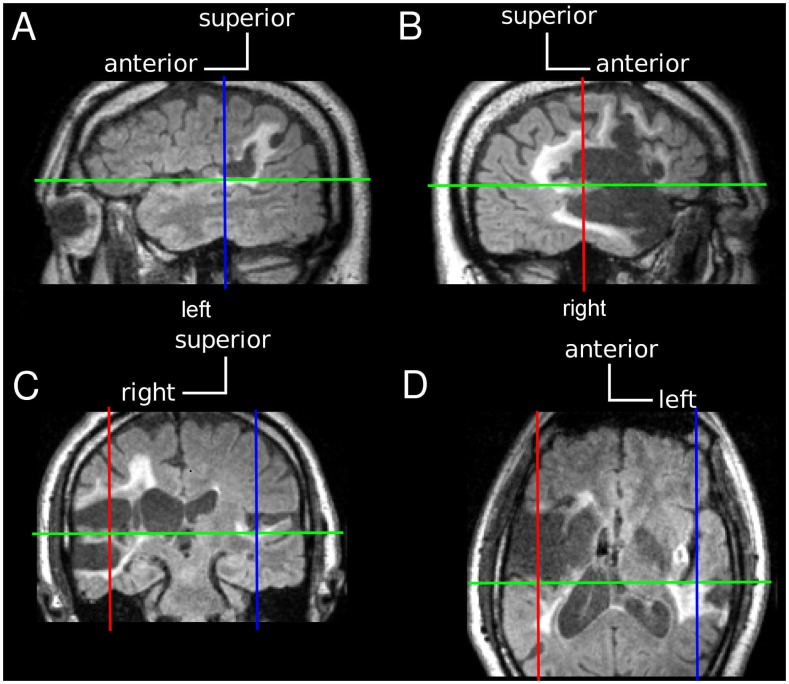
Case A1+ MRI FLAIR sequences. (**A,B**) Parasagittal sections through the left and right hemispheres. Left TG is atrophic and right TG is replaced by encephalomalacia (low signal intensity). Ischemic demyelination and retrograde degeneration within adjacent white matter regions appear as areas of high signal intensity. (**C**) Coronal section through the mid-portion of left and right TG and STG. (**D**) Horizontal section through left and right TG and STG. See text for image acquisition parameters.

Multi-planar MRI sections were acquired four months after the present psychoacoustic experiments using a Siemens TIM Trio 3T. Lesion localization was analyzed on fluid-attenuated inversion-recovery (FLAIR) sequences. Contiguous sections were 1.0 mm-thick with an in-plane resolution of 0.94 mm^2^ (TE = 494 ms, TR = 6000 ms, IT = 2100 ms, flip angle = 120°). Selected parasagittal, coronal, and horizontal MRI sections through superior temporal cortex are illustrated in Fig. 2. Abnormal FLAIR signal involves all of the right TG, all of left TG, all or almost all of right superior temporal gyrus (STG), a portion of left STG posterior to TG, and underlying white matter, including the geniculo-temporal radiation. The right-hemisphere lesion extends into adjacent frontal, temporal, and parietal areas; the smaller left-hemisphere lesion extends into adjacent temporal and parietal areas. Comparison with previous MRI sections through superior temporal cortex found no change [4].

At the time of the present experiments, Case A1+ reported difficulty perceiving speech - especially in noisy environments and on the telephone – as well as difficulty localizing sounds. He was alert, attentive, and without complaints throughout the psychoacoustic experiments.

#### Normal controls

Eleven age-matched right-handed adults (7 female, 4 male) participated as normal controls (median age = 41 years, range = 32–50 years). None reported a history of neurological disease or hearing impairment, and none were formally-trained musicians or actively performing music.

### Stimulus Delivery and Data Collection

Participants sat in a double-walled sound-attenuated booth and faced a computer monitor on which instructions, visual cues, and feedback were given. Participants entered their responses using a computer keyboard. All stimuli, except for pure tone audiometry in normal control subjects, were generated digitally using MATLAB (The Mathworks) and converted to analog waveforms by a LynxOne (LynxStudio) 24-bit soundcard with a sampling frequency of 32 kHz. The stimuli passed through programmable attenuators (TDT PA4, Tucker Davis Technologies) and headphone buffers (TDT HB6, Tucker Davis Technologies) before presentation to the subject via HD580 headphones (Sennheiser).

### Pure-tone Detection

A two-interval, two-alternative, forced-choice (2I-2AFC) paradigm with a 2-down, 1-up adaptive procedure was used to measure the minimum intensity Case A1+ needed to detect the presence of a 1-kHz, 500-ms pure tone with a response accuracy of 70.7% [17]. Each interval’s occurrence was indicated visually by one of two boxes on the computer screen labeled “1” and “2”; box 1 flashed during the first interval and box 2 during the second interval. The target tone (duration = 500 ms, 20-ms raised-cosine ramps) was randomly assigned to the first or second interval; no stimulus was present in the other interval. The threshold for each run was defined as the mean dB SPL of the last six turnaround points. Thresholds in normals were measured with an Interacoustics Diagnostics Audiometer (AD229e) and Telephonics headphones (TDH-39P) using a modified Hughson-Westlake procedure.

### Loudness Discrimination

On each trial, two 1-kHz pure tones were presented. Each tone had a duration of 500 ms and was gated on and off with 20-ms raised-cosine ramps. The two tones were separated by an inter-stimulus interval (ISI) of 200 ms. The same 2I-2AFC procedure used for pure-tone detection was used here. One tone was at the reference intensity (I = 65 dB SPL for normal controls, I = 40 dB SL for Case A1+). The intensity of the “test” tone (I+ΔI) differed slightly in intensity from the reference by adding a 1-kHz, in-phase tone to the reference tone. The order of the reference and test tones was randomized on each trial. Listeners judged whether the second tone was “louder” or “softer” than the first tone.

Intensity difference thresholds were expressed as ΔL = 10log10[(I+ΔI)/I)] [18]. A 2I-2AFC, 2-down, 1-up adaptive procedure tracked the 70.7% correct point on the psychometric function. In order to maximize the number of observations made near threshold, step size was decreased serially (ΔL = 2.45, 1.48, 0.54 dB) over the course of the run. Threshold for each run was defined as the mean ΔL of the last six turnaround points after the smallest step size had been reached.

Normal listeners participated in three left-ear runs and three right-ear runs. Contralateral noise at a level (per equivalent rectangular bandwidth) of 20 dB below the target was presented in order to prevent the use of the contralateral ear in performing the task [19]. The start ear was pseudorandomized across subjects such that an equal number of subjects started in each ear. Subjects performed three or more practice runs until performance plateaued.

Case A1+ participated in six runs for each ear. We did not present contralateral masking noise. Blocks were counter-balanced by ear using an ABBA paradigm: Right ear, left ear, left ear, right ear, with three runs per block. The start ear was randomly chosen. Case A1+ performed three or more practice runs until performance plateaued.

## Results

### Intensity Thresholds for Pure-tone Detection

**Figure 3:**
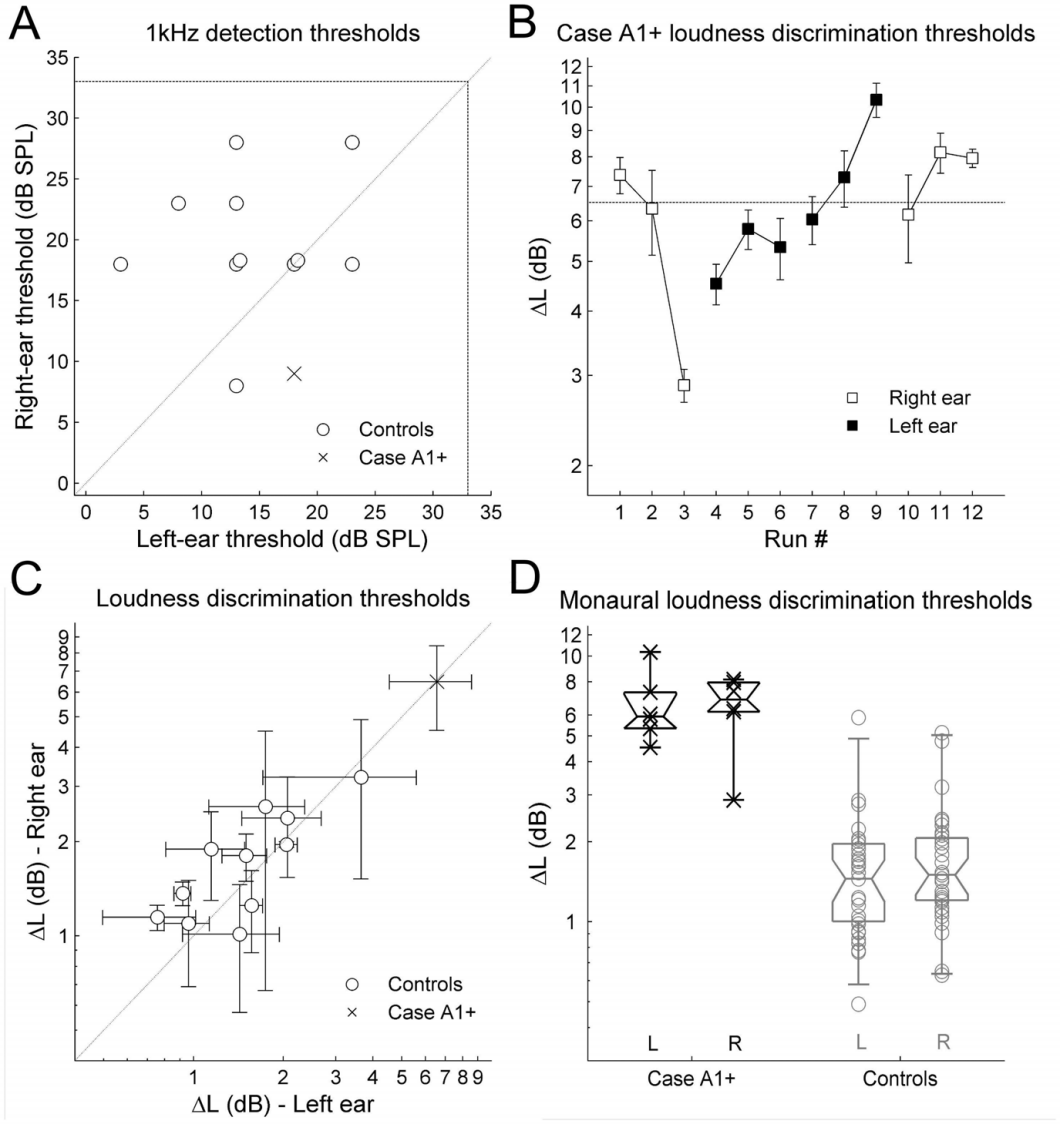
Comarison of detection and discrimination thresholds for Case A1+ vs. controls.

Fig. 3A shows pure-tone detection thresholds for Case A1+ and the eleven normal controls. All were within normal clinical limits [defined as 33 dB SPL at 1 kHz]. For Case A1+, the detection threshold in the left ear, which is contralateral to the larger lesion, was 18 dB SPL; the detection threshold in the right ear was 9 dB SPL. His left-ear threshold was within one standard deviation (SD) of the mean (M) of the control population (M ± SD = 14.4±6.0 dB SPL). His right-ear threshold was within 2 SDs of the control mean (M ± SD = 19.8±5.6 dB SPL), though a Wilcoxian signed-rank test indicated that Case A1+'s threshold was lower than our normal control population (signed-rank = 1, p = 0.002). The absolute difference between the left and right ears in Case A1+ (9 dB) was within a half SD of the control mean (7.3±5.6 dB).

### Intensity Thresholds for Pure-tone Loudness Discrimination

Fig. 3B shows Case A1+ ΔL thresholds for individual runs in chronological order from the first run through the last. A Kruskal-Wallis test found no significant order effect across blocks for Case A1+ (χ^2^ = 5.21, p = 0.16).

Fig. 3C shows ΔL thresholds (M ± SD) for left- and right-ear loudness discrimination. Fig. 3D shows the individual run data; box-and-whisker plots give the population median, interquartile range, and estimated 95% confidence interval. For Case A1+, ΔL thresholds averaged across the six runs for each ear were 6.5±2.1 dB in the left and 6.5±1.9 dB in the right. For normal controls, ΔL thresholds were 1.6±0.22 dB in the left ear and 1.7±0.19 dB in the right ear. In each ear, Case A1+'s median ΔL was above – with only one ΔL measurement within – the 95% confidence interval of the control population. A Mann-Whitney U-test using the ΔL for each run as the dependent variable confirmed that Case A1+ thresholds were significantly higher than those of normals in each ear [left and right ear: U = 216, p<0.00001].

Inspection of Figs. 3B and 3C suggests no significant left-right ear differences. A Mann-Whitney U Test using the threshold from each run confirmed this for Case A1+ (U = 34, p = 0.48, N = 6 per ear) and for controls (U = 1031.5, p = 0.35, N = 33 per ear).

## Discussion

The results support our first working hypothesis: pure-tone ΔL thresholds for louder-softer loudness judgments were 5 dB greater in Case A1+ than normals. Contrary to our second *a priori* hypothesis, ΔL thresholds were not significantly greater in the left ear despite his larger right hemisphere lesion. Finally, consistent with our third working hypothesis, intensity thresholds for tone detection were within normal limits at all frequencies tested, including the same frequency (1 kHz) used to test tone loudness discrimination. These findings support the claim that brain mechanisms mediating auditory sensation and perception are neurologically dissociable. In addition, the fact that Case A1+ was able to perform louder-softer judgments, albeit at much higher ΔL thresholds, indicates that auditory structures spared by his strokes – specifically left anterior auditory association cortex and/or the auditory brainstem – can mediate coarse loudness perception.

It should be noted here that the test conditions for Case A1+ and normals were not identical (see Methods). However, based on our review of relevant literature on the differences between one- and two-interval forced-choice tasks [20] as well as the effects of contralateral masking noise [21] and reference intensity level [7] on loudness discrimination thresholds, it is unlikely the differences affect our conclusions. In fact, the fact that we used contralateral masking noise in our control population and not in Case A1+ may have underestimated his deficit. It is also unlikely that the observed deficits in Case A1+ are attributable to non-modality specific effects of his lesions. First, we did not observe signs of fatigue during testing, and there was no significant order effect across blocks. Second, his intensity thresholds for pure-tone detection were normal and were measured with an adaptive procedure that was as demanding as the one used to measure loudness discrimination. Lastly, previous psychophysical measurements demonstrated that both duration discrimination thresholds (for long-duration pure tones) and vibrotactile intensity discrimination thresholds were only slightly impaired [6].

### Lesion-effect Studies in Humans

While our single-case study establishes an existence proof for an auditory-cortex role in loudness perception, the generalizability of our findings must be assessed in the broader context provided by previous rare cases with bilateral auditory cortex lesions as well as cases with unilaterallesion cases studied with suitable psychoacoustic methods (summarized in Table 1).

**Table 1:** Summary of human lesion effects on loudness perception.

	Monaural -Left	Monaural -Right	Binaural
Left lesions – including TG			
Milner [20], N = 16	n/a	n/a	O
Swisher [21], N = 8	o	O	O
Hodgson [22], N = 1	o	++	n/a
Baran et al. [23], N = 1	o	++	n/a
Left lesions – not including TG			
Swisher [21], N = 10	o	O	O
Right lesions – including TG			
Milner [20], N = 11	n/a	n/a	+
Swisher [21], N = 18	o	o	O
Bilateral lesions			
Jerger et al. [24], N = 1	+++	+	n/a
Jerger et al. [25], N = 1	++	+	n/a
Case A1+	+++	+++	n/a

O = no deficit, + = mildly impaired, ++ = moderately impaired, +++ = severely impaired. The extent of damage to TG is unknown for Jerger et al. [24]. The lesions in Jerger et al.’s case [25] extended into TG bilaterally.

Most patients with unilateral lesions showed little or no deficit for intensity-discrimination thresholds [22], [23] (but see [24], [25]), somewhat irrespective of whether the insult broached TG. Conversely, both patients with bilateral lesions to the superior temporal cortex had clear deficits [26], [27], consistent with the results from the present study. The single case in whom lesions were localized precisely showed bilateral involvement of TG and posterior STG [27]. Given the conflicting results of unilateral lesion studies in temporal lobectomy patients, and the conclusions from Baru and Karaseva’s review of the Russian and German literature [28], we conclude that unilateral A1 and AA lesions have little to no effect on either binaural or contralesional loudness perception.

### Lesion-effect Studies in Non-human Primates

We found only two Old-World monkey studies that examined the effects of auditory cortex lesions on intensity processing [29], [30] in Old World monkeys. In one [29], Rhesus macaques were trained to detect when a 1-kHz tone decreased in intensity from 80 dB to 60 dB SPL before bilateral ablation of the superior temporal plane including primary auditory cortex. After ablation, the subject did not reach the criterion level of performance of 90% correct in 25 sessions, after which the comparison intensity was decreased to 40 dB, for which the subject achieved criterion after 55 sessions. In the other [30]. Japanese macaques judged whether a three-tone sequence was louder or softer than a three-tone standard sequence presented at 65 dB SPL in quasi-free field. The monkey with near complete bilateral ablations of A1 and AA had elevated jnd's, while none of three monkeys with extensive unilateral A1 and AA lesions showed a deficit.

### Chronic vs. Acute Lesions

The aforementioned lesion-effect studies all examined the impact of chronic lesions of auditory cortex on sound detection or intensity discrimination, where long-term compensatory mechanisms are likely to have occurred. Indeed, Case A1+'s second infarct left him profoundly deaf for at least a month, after which he slowly recovered the ability to detect high- and moderate-intensity sounds. In contrast, acute lesion studies (e.g., using inhibitory agonists or reversible cooling) have the unique ability to reveal the brain areas which normally support a given function whilst ruling out compensatory reorganization. Such studies, mostly carried out in non-primate mammals, have produced mixed results regarding whether auditory cortex is normally involved in the detection and/or discrimination of sound [31]–[34], although it seems likely that acute inactivations of auditory cortex can produce disruption of both frequency discriminaticomon and sound localization [35], [36] as well as more complex functions [37]. In any case, the fact that Case A1+'s deficit in intensity discrimination persists years after his last infarct suggests that in healthy humans, fine-grained intensity processing is (i) supported by auditory cortex and (ii) cannot be completely restored by post-infarct compensatory mechanisms, although such mechanisms could play a role in restoring sound detection and coarse loudness discrimination.

### Neuroimaging Studies of Intensity Processing

The coarse neural representation of sound level has been investigated extensively using auditory-evoked potentials, PET, and fMRI [8]–[11], [38]–[49]. These studies have consistently demonstrated that increases in intensity correlates with increased activity in auditory cortex. The most relevant study for the present discussion measured sound-evoked BOLD activation as a function of intensity while subjects detected occasional changes in duration [8]. Increases in sound level produced non-linear increases in both magnitude and spatial extent of activation, with stronger increases in TG (vs. STG) and contralateral (vs. ipsilateral) to the ear of stimulation. Other fMRI studies have also demonstrated high correlations between intensity and TG activation, particularly its posterior-medial portion [10], [38], the presumed core of human auditory cortex [12], [13]. The one study which examined subcortical structures also found activation which increased with increasing intensity [10].

## Conclusions

The present findings in Case A1+, in line with previous neuroimaging and neuophysiological studies not reviewed here [50]–[55], advance the claim that auditory cortex plays a critical role in basic auditory functions, particularly with respect to the fine-grained analysis of spectral information [56]. Although auditory cortex plays a critical role in fine-grained loudness discrimination, sensation *per se* and course loudness discrimination in the chronic state remain after complete bilateral lesions of A1 and near-compete bilateral lesions of AA. We therefore hypothesize that intensity processing is organized hierarchically: the auditory cortex (most likely A1) is necessary for fine-grained loudness discrimination (though an explanation in terms of top-down effects due to loss of descending projections from the auditory cortex to subcortical structures cannot be ruled out, see e.g. [57], [58]), while the auditory brainstem may be sufficient for detection of sound after cortical insult. It remains unclear whether the auditory brainstem, auditory association cortex, or both are sufficient for coarse loudness discrimination and subsequent response mapping after bilateral auditory cortex lesions.

As a consequence of his chronic bilateral A1 and AA infarcts, Case A1+ needed tones to be twice as loud to discriminate increases from decreases in loudness. Given the importance of musical and speech dynamics to aesthetics, prosody, and semantic processing, these deficits in basic auditory functions would likely have profound effects at cognitive and emotional levels [59], [60].
